# Zinc finger myeloid Nervy DEAF-1 type (ZMYND) domain containing proteins exert molecular interactions to implicate in carcinogenesis

**DOI:** 10.1007/s12672-022-00597-9

**Published:** 2022-12-15

**Authors:** Longji Wu, Jing Huang, Pankaj Trivedi, Xuerong Sun, Hongbing Yu, Zhiwei He, Xiangning Zhang

**Affiliations:** 1 Department of Pathophysiology, School of Basic Medicine, Guangdong Provincial Key Laboratory of Medical Molecular Diagnostics, Guangdong Medical University, Songshan Lake Scientific and Industrial Park, Dongguan, 523808 Guangdong People’s Republic of China; 2 Chinese–American Tumor Institute, Guangdong Provincial Key Laboratory of Medical Molecular Diagnostics, Guangdong Medical University, Dongguan, Guangdong People’s Republic of China; 3 Institute of Aging, Guangdong Provincial Key Laboratory of Medical Molecular Diagnostics, Guangdong Medical University, Dongguan, Guangdong People’s Republic of China; 4 Department of Experimental Medicine, La Sapienza University, Rome, Italy; 5 Present Address: Institute of Modern Biology, Nanjing University, Nanjing, Jiangsu China

**Keywords:** Deformed epidermal auto-regulatory factor-1, Zinc finger myeloid Nervy DEAF-1 type containing motif, Myeloid translocation gene on chromosome 8 (MTG8), Beta catenin in lung cancer, Programmed cell death 2, Transcription repression, Histone modification, Tumor suppression, Leukemia, Nasopharyngeal carcinoma

## Abstract

Morphogenesis and organogenesis in the low organisms have been found to be modulated by a number of proteins, and one of such factor, deformed epidermal auto-regulatory factor-1 (DEAF-1) has been initially identified in Drosophila. The mammalian homologue of DEAF-1 and structurally related proteins have been identified, and they formed a family with over 20 members. The factors regulate gene expression through association with co-repressors, recognition of genomic marker, to exert histone modification by catalyze addition of some chemical groups to certain amino acid residues on histone and non-histone proteins, and degradation host proteins, so as to regulate cell cycle progression and execution of cell death. The formation of fused genes during chromosomal translocation, exemplified with myeloid transforming gene on chromosome 8 (MTG8)/eight-to-twenty one translocation (ETO) /ZMYND2, MTG receptor 1 (MTGR1)/ZMYND3, MTG on chromosome 16/MTGR2/ZMYND4 and BS69/ZMYND11 contributes to malignant transformation. Other anomaly like copy number variation (CNV) of BS69/ZMYND11 and promoter hyper methylation of BLU/ZMYND10 has been noted in malignancies. It has been reported that when fusing with Runt-related transcription factor 1 (RUNX1), the binding of MTG8/ZMYND2 with co-repressors is disturbed, and silencing of BLU/ZMYND10 abrogates its ability to inhibition of cell cycle and promotion of apoptotic death. Further characterization of the implication of ZMYND proteins in carcinogenesis would enhance understanding of the mechanisms of occurrence and early diagnosis of tumors, and effective antitumor efficacy.

## Introduction

The proteins with the domain of zinc finger (zf)-myeloid-Nervy-deformed epidermal autoregulatory factor-1 (ZMYND) type was initially identified in low organisms like Drosophila melanogaster; the proteins, as exemplified with DEAF-1 known as suppressin and later ZMYND-5, is a binding partner of the autoregulatory enhancer upstream of coding portion of the Deformed (Dfd) Hox gene [1, 2].

In Drosophila melanogaster, different identities of cells along the anterior–posterior axes contribute to the pathway components of the homeotic complex (HOM-C) genes. The coding homeoproteins recognize similar DNA sequence with identical binding affinities and one of them recognizes a variety of DNA sequences, almost all of the proteins contain a core sequence TAAT [3, 4]. Identification of the specific downstream target genes is essential for understanding specification of HOM-C in axial patterning, it is therefore necessary to identify controlled target genes downstream of the specific homeoproteins.

The homeotic gene Deformed has been recognized as a target of HOM-C regulated through autoregulatory activation during embryonic development. Several autoregulatory elements have been identified; they possess the activities with Deformed-responsive maxillary enhancers [2, 5]. It has been described that the 664-bp Deformed Response Element (1.28 DRE) in Drosophila, is potentially targeted by Deformed [2], directing maxillary-specific expression of a reporter gene in transgenic embryos. In vitro, it has been found that the 1.28 DRE contains binding sites for Deformed and deformed autoregulaotry factor-1 (DEAF-1), and was recognized as a novel protein from Drosophila nuclear extracts which binds specifically to a site in this region. The protein termed DEAF-1 (Deformed epidermal autoregulatory factor-1) has been shown to contain three conserved domains [2]. One of them includes a cysteine repeat motif that is similar with a motif found in Drosophila Nervy and the human myeloid transforming gene 8 (MTG8) encoded proto-oncoprotein, and another matches a region of Trithorax encoded by Drosophila genome. Site specific mutations in the response element improve binding to DEAF-1 in vitro, and resulted in increased the embryonic expression of DEAF-1. These regions that bound by DEAF-1 are not required for enhancer function [6].

The homeoproteins are activated in head segments of Drosophila embryo [2]. Several TTCG motifs within the portion of the Dfd autoregulatory region are recognized by DEAF-1. In addition, DEAF-1 associates with similar motifs within a Dfd response element (DRE) to enhance maxillary gene expression during embryogenesis [7]. Subsequently, a number of proteins with structural similarity are discovered; they are highly conserved among species and contain one to several functional domains, with MYND in common, and perform different activities like recognition of genomic marks, modifying arginine and lysine residues on histones, repressing gene transcription through molecular interaction and protein degradation; they are implicated in innate immune response [8] in Drosophila, as well as tumor suppression, and tumorigenesis.

It has been reported that a member of ZMYND protein family, ZMYND10, plays a role in the ciliary formation during organogenesis. The core axoneme in Drosophila a central-pair microtubule apparatus is surrounded by nine peripheral outer doublet microtubules, whereas motile embryonic node monocilia lack apparatus of the central-pair [9]. Studies in ciliated organisms, including Chlamydomonas, and trypanosomes [10] have shown that the axoneme facilitates microtubules (inner-dynein-arm [IDA] and outer-dynein-arm [ODA] bending via the dynein motors’ ability. In *Paramecium tetraurelia*, the absence of ZMYND10 causes the abnormal localization of IFT43, a intraflagellar transport (IFT) protein along cilia. These results suggest that ZMYND10 involves regulating ciliary function and IFT [11].

**Fig. 1:**
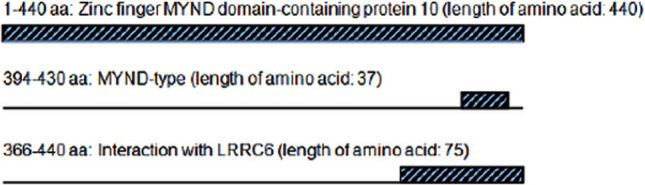
Scheme of linear structure with motif composition of ZMYND10 protein. A MYND domain with 37 residues capable of binding zinc, conserved among ZMYND family proteins is located at the carboxylterminus, spanning the amino residues 394–430. A 75-residue motif is responsible for interaction with leucine repeat rich component 6 (LRRC6) mutations within which lead to primary cilia dyskinesia. The figure was adapted from [13], reused with permission from Atlas in Genetics and Cytogenetics of Oncology and Haematology

In primary ciliary dyskinesia (PCD [MIM 244400]) affected families, biallelic variants in ZMYND10 (MIM 607070; GenBank Accession Number NM_015896.2) were found to be shared by affected individuals from each of families with biallelic variants of interest [12]. Familial study of PCD reveals mutations in ZMYND10 and LRRC6 which interact. Certain ZMYND10 and LRRC6 mutations abrogate the interaction between CS domain in LRRC6 and C-terminal domain of ZMYND10 (Fig. 1) [13]. The two molecules are colocalized with the centriole markers SAS6 and PCM1. Mutations in ZMYND10 result in the absence of DNAH5 and DNALI1 as the axonemal protein components, seen in respiratory cilia [14].

Study in human cancer cells suggests that ZMYND10 behaves as a signaling molecule, and, combined with the findings of its downregulation in tumor tissues and cancer cells. It is regarded as a candidate tumor suppressor [15–17]. The tumorigenic and tumor suppressive of ZMYND proteins have been observed; their functions are exerted through regulation of gene expression, and cell cycle progression. Oncogenic as well as tumor suppressive potential of ZMYND proteins are discussed in details in the present paper. SMYD proteins and AML1-ETO/MTG8/ZMYND2, MTG16/ZMYND3, MTG R1/ZMYND4, RACK7/ZMYND8, and BS69/ZMYND11 are well known to be implicated in carcinogenesis though recruitment and modification of target proteins.

**Fig. 2:**
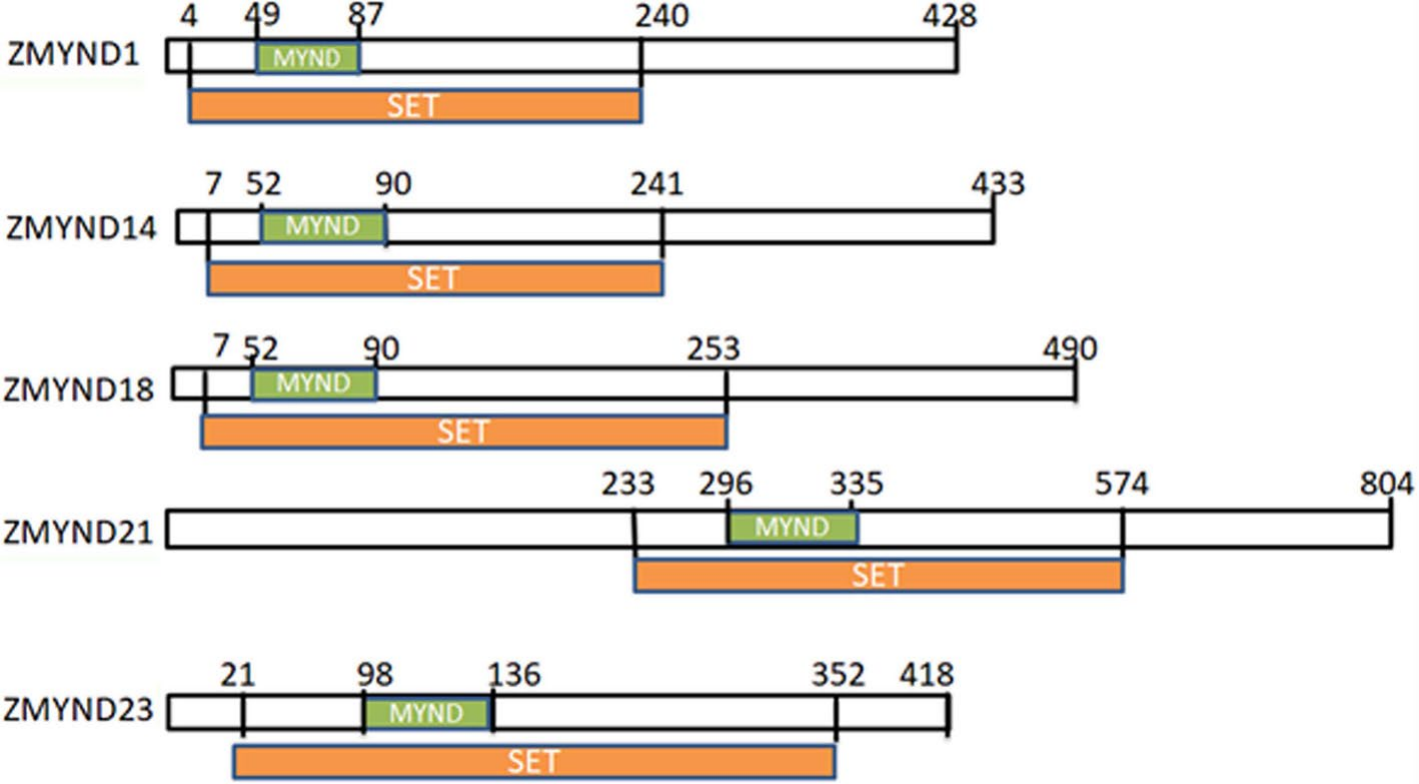
Scheme of linear structure of ZMYND proteins with SET and MYND domains. The MYND domains are intervened in the longer SET domains which are separated by inserted fragments. The numbers are defined as the position of the amino acid residues within the molecules

## ZMYND proteins that modifying histone marks to regulate gene expression: ZMYND1, ZMYND14, ZMYND18, ZMYND21, ZMYND23

### Basic structure of SMYD family proteins

A group of proteins concurrently contain domains Suppressor of variegation [Su (var)), Enhancer of zeste, Trithorax (SET) and MYND within their linear structure. The SET domain is a fragment with 130 to 140 amino acid residues, as an evolutionarily well conserved sequence motif; it was initially described in the homologues Su(var) 3-9, Enhancer-of-zeste and Trithorax in Drosophila [18, 19]. Up to now, five members have been identified. They are termed SMYD proteins, forming a family with five members, that is SMYD1, 2, 3, 4, and 5. These proteins also fall into the family of ZMYND with nomenclature of SMYD1/ZMYND18/BOP, SMYD2/ZMYND14/, SMYD3/ZMYND1, together with SMYD4/ZMYND21, SMYD5/ZMYND23/retinod acid induced 15(RAI15). It has been known that SMYD proteins function to recruit lysine residues on the histones, and catalyze methylation as lysine methyltransferase (KMT) of histone and non-histone proteins, SMYD1, 2, 3 are alternatively called KMT3D, KMT3C, and KMT3E.

The core SET domain of SMYD protein family with catalytic activity is split into two parts by the intervention of the MYND domain [20]; and the MYND domain within remaining SET domain (C-SET) is separated by an intermediate linker sequence acting as a binding cofactor to be implicated in protein–protein interactions (see Fig. 2) [21]. Among member of the SMYD family, SMYD3 contains the domains with catalytic importance, forming a deep and narrow pocket for substrate binding, apart from the classical SET domain [21]. MYND domain is associated with the interacting protein partners through PXLXP motifs and stimulates the catalytic activity of SMYD proteins [22, 23]. The SET domain is split into two segments (the S-sequence and a core SET domain) by the MYND domain. All members of this family have post-SET and SET-I domains, while the C-terminal domain (CTD) is found in only SMYD1-4 [24, 25] (Table 1). The tetratricopeptide repeats (TPR) is contained only in the amino(N)-terminus of SMYD4, as with SMYD5/ZMYND23/RAI15, the domain is absent in N-terminus and in the carboxylterminal domain (CTD). The motif is important for interaction with the heat shock protein 90 and amino acid residues on peptide tails of histones [26] (Table 1).

**Table 1:** Distribution of functional domains in members of SMYD family protein

Protein/domains	TPR	S	MYND	SET-1	Post-SET	CTD	Length^a^
SMYD1	–	+	+	+	+	+	490
SMYD2	–	+	+	+	+	+	433
SMYD3	–	+	+	+	+	+	428
SMYD4	+	+	+	+	+	+	804
SMYD5	–	+	+	+	+	–	418

The rightest column is the full length of each protein with amino acid residues. The present data is adapted from [26]

^a^The length is defined as number of amino acid residues

### Catalytic activities to methylate histone exerted by SMYD family proteins.

A methyltransferase, Enhancer of zeste homolog-2 (EZH2) is the catalytic subunit of polycomb repressive complex-2 (PRC2). A sequence within the molecule is similar with enhancer of zeste [27, 28]. The PRC2-related proteins transcriptionally silence tumor suppressor genes (TSGs) by catalyzing trimethylation of a repressive mark in lysine 27 of histone H3 (H3K27). Perturbations of PRC2 activity have been found in many cancers and pharmacological inhibitors of PRC2 are currently under evaluation [29]. Aberrant hypermethylation and enrichment of de novo methylation at PRC2-related gene loci are commonly found in nasopharyngeal carcinoma (NPC) [30], a malignant tumor endemic in southern China, Southeast Asia, Alaska, Greenland and Maghrebian countries in North Africa. Overexpression of EZH2 of has been found in 46–80% of NPC tumor and is associated with advanced disease stage [30–32]. The effect of targeting PRC2 activity on the growth inhibitory effect of platinum-based chemotherapy also warrants investigation given the important role of chemotherapy in the treatment of NPC [33, 34].

As suggested by composition of motifs, as well as the generic names of some SMYD family proteins, they are members of KMT family, functioning to catalyze methylation to residues of lysine 4 or 36 on histone 3 (H3K4, H3K36) or lysine 20 on histone 4 (H4K20), also lysine residues on non-histone proteins like tumor suppressor p53 (TP53) or heat shock protein 90 (HSP90). H3K4 and H3K36 in form of methylation modification are active marks, while H4K20, like H3K9 and H3K27, represses gene transcription on the status of some forms of methylation [35]. SMYD1/ZMYND18/ KMT3D/BOP plays a key modulating role in the embryonic heart development via inhibiting differentiation of cardiomyocyte and malformation of the right ventricle, through regulation of gene expression via methylation of H3K4 in a locus-specific manner [36, 37]. The catalytic substrate of SMYD2/ZMYND14/KMT3C is H3K36 on the peptide tail stretching from the core of nucleosome, as an active mark for transcription. In contrast to the tissue specificity of heart and skeletal muscles of SMYD1, its expression has been identified in different tissues [38, 39], and it behaves as an oncoprotein to repress the tumor suppression exerted by p53 by modifying lysine 370 by methylation [40] and that of retinoblastoma through methylation on lysine 860 [41]. In the pancreas, SMYD2 exerts its function of oncoprotein in the cytoplasm by methylating of MAPKAPK3 on its lysine 355. MAPKAPK3 is a kinase that participates in the Ras signaling pathway and involves stress and inflammation responses [42]. SMYD3 was originally identified as a methylase of H3K4 and it is involved in the proliferation of colon and liver cancer cells [43]. SMYD3 also catalyzes methylation of lysine 5 of histone 4 (H4K5) [44]. A study in breast cancer suggests that re-expression of SMYD4 suppresses growth of tumor cells and inhibits formation of xenografted tumor in nude mice. Microarray studies revealed that platelet-derived growth factor receptor alpha polypeptide (Pdgfr-alpha) is highly expressed in tumor cells. Re-expression of SMYD4 has been shown to significantly reduce the expression of Pdgfr-alpha in tumor cells. In some specimens of human breast cancer tested reverse transcription-PCR results revealed that SMYD4 expression was totally silenced. The finding revealed that SMYD4 exerts tumor suppression in breast cancer through the partial inhibitory effect of PDGFa [45]. SMYD5 regulates pro-inflammation genes through trimethylating H4K20 and interacting with the NCoR corepressor complex and acts as a repression checkpoint restricting the expression of toll like receptor 4 (TLR4) in macrophages [45];

It has been found that in addition to coding region of genes, H3K36me3 is enriched at promoters in primary cells. SMYD5 has been identified to be recruited to chromatin by RNA polymerase II, as a methyltransferase catalyzing H3K36me3 at promoters. It has been reported that overexpression of full-length Smyd5, but not the C-terminal domain-truncated Smyd5, restores H3K36me3 at promoters in Smyd5 knockout cells. Furthermore, elevated Smyd5 expression contributes to tumorigenesis in liver hepatocellular carcinoma [46].

### The activity of SMYD1 in muscle development

SMYD1 plays a crucial role in heart development. It has been described that SMYD1 catalyzes the methylation of H3K4 and non-histone proteins such as skeletal and heart muscle-specific variant of nascent polypeptide-associated complex (skNAC). skNAC is exclusively found in striated muscle cells. The binding of skNAC with SMYD1 suggests the activity of the complex in regulation of transcription. It has been reported that transcriptional control exerted by skNAC-SMYD1 was exerted by histone modification, in relationto the catalytic activities of SMYD1 including bi- and trimethylation, and potentially acetylation. A series of target genes have been identified; they are genes encoding regulators of inflammation, cellular metabolism, and cell migration [47]. Defects of morphogenesis such as defects of cardiac outflow tract and pancreatic and diaphragmic malformations can be caused by damage of GATA6, which is a pioneer factor in cardiac development, regulating SMYD1 that activates heart- and neural crest derivatives-expressed protein 2(HAND2), that orchestrates outflow tract formation[48]. When apoptosis is triggered in cultured rat myoblasts (H9c2) with doxorubicin (DOX) at 1 or 5 μM for 24 or 48 h, markers of oxidative stress, apoptosis and expression of proteins involved in epigenetic processes were assessed. IT has been shown that DOX treatment causes severe cell death by apoptosis associated with cellular oxidative stress, and proteins involved in epigenetic processes, histone deacetylases (SIRT1 and HDAC2), histone lysine demethylases (KDM3A and LSD1) and SMYD1 were significantly regulated. Histone 3 acetylation in DOX-treated cells is significantly decreased [49].

### The implication of SMYD2 in the occurrence of cancer

Triple-negative breast cancer (TNBC), the type of the tumor in absence of expression of estrogen receptor (ER), progesterone receptor (PGR) and human epidermal growth factor receptor 2 (HER2) is a common and aggressive subtype of breast cancer with poorer prognosis and shortened survival duration. It has been shown that the level of SMYD2 in TNBC is correlated with tumor progression and poor prognostic outcome; and that inhibition of SMYD2 reverses malignant transformation [50].

SMYD2 functions to methylate histone marks H3K4, and H3K36, and activates expression of target genes through its interaction with corepressors [51, 52]. In addition to downregulation of p53 and Rb through methylation on their lysine residues to accelerate cell cycle progression [40, 41], SMYD2 also methylates heat shock protein 90 (HSP90) [32] and several sites in ERα to prevent its recruitment to the promoters of its target gene [51, 52].

Overexpression of SMYD2 has been reported in primary tumor samples of esophageal squamous cell carcinoma (ESCC) and pediatric acute lymphoblastic leukemia (ALL); the expression level is correlated with a poor prognosis and shortened survival of the patients [53, 54]. SMYD2 promotes the genesis of TNBC via activation of STAT3 and the p65 subunit of NF-κB, i.e. NF-κB3 [50]. It mediates methylation of histones to transcriptionally regulate the expression of cancer driver genes. Knockdown of SMYD2 and inhibition of SMYD2 with The specific inhibitor, AZ505 knocks down and inhibits SMYD2, and prevents the growth of TNBC cells xenografted in nude mice [50].

The important role of SMYD2 has been revealed by a growing number of studies in several types of cancer, including breast, liver, and gastric cancers, in addition to leukemia, and ESCC [53–59]. Overexpression of SMYD2 protein is observed in most primary tumor samples of ESCC, and in some ESCC cell lines. ESCC patients with overexpressed SMYD2 have a lower overall survival rate than those with low level of SMYD2 expression [53]. In comparison with normal individuals, mRNA levels of SMYD2/3/5 are significantly increased and those of SMYD1/4 are reduced in patients of breast cancer. SMYD2 mRNA expression level is associated with metastatic relapse in the relapse-free survival of breast cancer patients, indicating that SMYD2 acts as a potential biomarker and prognostic indicator for this cancer [55]. In addition, overexpression of SMYD2 has been observed in hepatocellular carcinoma (HCC) [56, 57], gastric cancer [58], pancreatic ductal adenocarcinoma (PDAC) [59], papillary thyroid carcinoma [60], and colon cancer [61]. In non-small-cell lung cancer (NSCLC), multiple nonhistone proteins are modified by SMYD2 mediated methylation, and effects on tumors are generated by the modified activity of signaling pathways, for example, MAPKAPK3 in PDAC and ALK [62]. In HCC and thyroid cancer, overexpression of SMYD2 is positively correlated with enlarged tumor size, more rapid lymphatic invasion, more potential tumor invasion, and higher TNM stage, as well as worsened overall rate of survival [57, 60, 63–66].

### The implication of SMYD3 in occurrence of cancers

SMYD3 was originally identified as methylase of the histone mark H3K4 [67]. It is involved in the proliferation of cells of colon and liver cancers and is also shown to catalyze methylation of H4K5 [68]. The discovery of SMYD3 and recognition of its importance on the survival of liver and colon cancer cells have stimulated a number of studies to establish its solid connection with cancer progression. It has been shown that overexpression of SMYD3 in different cancers is correlated to severe clinical implications in the patients, with significant reduction of overall survival and with accelerated disease progression towards more aggressive stages [68]. Overexpression of SMYD3 in breast cancer, with its impaired function leading to inhibition of cell cycle progression of breast cancer cells in vitro, through induction of cell cycle arrest of G0/G1 [69, 70]; mechanistically SMYD3 promotes breast carcinogenesis by transcriptionally activating oncogenes like WNT10B, N-myc, CrkL, RIZ and hTERT, cell cycle regulatory genes (cyclin G1, CDK2) and signal transduction pathway components (STAT1, MAP3K11, PIK3CB) [70]. Downregulation of the RIZ1 H3K9 methylase by SMYD3 has been linked liver cancer cell expansion in vitro, through DNA methylation of its promoter [71].

### The biological activities of SMYD4 and SMYD5

In addition to a role in muscle and cardiac development, and other pathophysiological conditions, SMYD4 has been defined as a tumor suppressor (review in [26]). An early study pointed to the role of SMYD4 as a tumor suppressor in breast cancer. Subsequently, it has been demonstrated that re-expression of Smyd4 (in breast tumour cells in which its expression had previously been suppressed) inhibited breast tumor cell and anchorage-independent growth [72].

SMYD5 has also been known for its methyltransferase activity; it mediates H4K20me3 marks at DNA sequences with LINE/LTR repeat [73]. By silencing lineage-specific gene expression, SMYD5 promotes mouse embryonic stem (ES) cell self-renewal; in this case, SMYD5 is recruited by LINE- and LTR-repetitive DNA elements to the vicinity of differentiation genes and they were maintained silenced due to the deposition of H4K20me3 marks. Heterochromatin formation and chromosome condensation are regulated in part by SMYD5 and silencing of endogenous retroviruses (ERV) in ES cells is mediated by H4K20me3 marks and interacting with chromatin repressors heterochromatin protein 1 (HP1) [74]. A role for histone-modifying enzymes mediated by The ERV-silencing properties of the H3K9 methyltransferase ESET is implicated during differentiation in heterochromatin formation and silencing of repetitive DNA elements. It has been shown that H4K20me3 is deposited at the promoters of a subset of these genes by the histone methyltransferase SMYD5 through its association with co-repressor N-CoR [74]. SMYD5 has been reported to be involved in the active basal repression of Toll-like receptor (TLR)4-responsive promoters in macrophages, by catalyzing trimethylation of H4K20me3. In zebrafish, it has been claimed that SMYD5 is a key regulator of both primitive and definitive hematopoiesis [75].

## ZMYND proteins that complex with co-repressors to regulate gene transcription: ZMYND2, ZMYND3 and ZMYND4 and ZMYND5

### The implication of molecular interaction of CBFA proteins in leukemogenesis

The proteins ZMYND2, 3, 4 were initially identified as the coding products synthesized by the fused genes formed by chromosomal aberrations during the genesis of some hematopoietic neoplasms, for example, acute myeloid leukemia (AML). Among these, ZMYND2 is encoded by Eight-Twenty-One (ETO)- myeloid translocation gene on chromosome 8 (MTG8) [76, 77]. Proteins ZMYND2, 3, 4 are alternatively termed CBFA2T1, CBFA2T2, and CBFA2T3, or RUNX1 Translocation partners 1, 2, and 3 (RUNX1T1, RUNX1T2, RUNX1T3). The nomenclature suggests that the genes are fused with transcription factor RUNX1 on chromosomal translocation, similar with the most common malignancy associated karyotype, t (8:21) seen in AML.

The structure of proteins ZMYND 2–4, is characterized by the presence of four conserved domains within the molecule, named Nervy homology regions 1–4 (NHR1–4) because of their sequence homology with that of the Drosophila protein Nervy (review in [77-79]). Two of these domains, NHR2 and NHR4, carry information for distinct but an altered histone code at target sites of hematopoietic gene [80]. All four NHRs interact with a wide range of regulatory proteins. The NHR1 region, sharing a homologous domain to TATA binding protein-associated factors (TAFs) [81], it involves transcriptional inhibition through displacement of P300/CREB-binding protein (CBP) coactivators [82, 83]. During leukemogenesis NHR2 oligomerizes with corepressors like the N-CoR/silencing mediator of retinoic acid and thyroid hormone receptor (SMRT), mSin3a, and histone deacetylases (HDACs) [26, 84].

NHR3 contains a coiled-coil structure to recruit transcriptional factors and NHR4 has two zinc-finger domains shown to recruit N-CoR/SMRT/HDAC transcriptional repressors [85]. Zinc finger MYND region in their carboxyltermus is referred to NHR4 in some reports [26]. The proteins are hence defined as members of ZMYND family. The motif composition of molecules in this group is summarized in Table 2.

**Table 2:** Motif composition of Nervy homolog regions containing proteins of ZMYND family

Proteins /parameters	ZMYND2/ RUNX1T1**	ZMYND3/ RUNX1T2 **	ZMYND4/ RUNX1T3	ZMYND5
Full length*	577	575	653	565
TAFH/NHR1	95–184	86–175	173–262	–
NHR2 domain like	310–376	302–368	379–445	–
NHR2	–	–	394–412	–
NHR3:	–	–	485–533	–
SAND	–	–	–	200–272
MYND/HNR4	488–524	478–514	556–592	504–540

^*^All number in the chart are defined as the position of amino acid residues; **isoform A of the these encoded proteins are entered comparison. The data were retrieved from https://www.ncbi.nlm.nih.gov/protein/ or https://www.ncbi.nlm.nih.gov/gene. TAFH: homology to TAF

The CBFA 2T1/RUNX1T1 gene is unexpressed or is expressed at a low level in normal hematopoietic cells [86]. The nonhomologous balanced translocation t(8;21)(q22;q22), involving fragment reciprocation between the chromosomes 8 and 21,with fusion of genes RUNX1-RUNX1T1 [87]. The translocation t(8; 21)(q22;q22) is the most common cytogenetic aberration in de novo AML [88]. It has been seen in 4%–12% of adult and 12–30% of pediatric de novo reported cases of AML and is the most common malignancy related karyotype in human leukemias [89]. RUNX1T1 is transcriptionally inactive. In turn, RUNX1-RUNX1T1 gene encodes a fusion protein termed myeloid translocation gene on chromosome 8 (MTG8) with the properties with transcription repression. It is believed that the translocation t(8; 21)(q22;q22) coding for a protein plays a critical role in the initiation of leukemogenesis in hematopoietic progenitor/stem cells [90, 91].

The product encoded by fused genes formed by the chromosomal translocation of (RUNX1)-/RUNX1T1/Eight-Twenty-One (ETO), and generates the RUNX1-ETO oncoprotein [92]. The cytogenetic aberration occurs during genesis of acute leukemia and other types of the lymphohematopoietic neoplasms and the fused gene encoded protein is highly expressed in the malignancies [93]. The chromosomal translocation reactivates RUNX1 and contributes to the development of leukemia. In addition, the absence of RUNX1T1 gene expression in leukemia cells obscures the structural and functional organization analysis of the RUNX1-RUNX1T1 fusion gene.

ZMYND 2 is encoded by ETO, also known as MTG8. Physiologically, ZMYND2 functions to repress gene transcription through interaction with co-repressor complexes, like nuclear corepressor (N-CoR) or mammalian Sin3A.

During t (8; 21)(q22;q22) the capacity of ZMYND2 to bind a repressor SIN3A is pertubed in this context, and leukemogenesis is achieved as a result [92, 93]. We have recently reported that another member of ZMYND family, ZMYND10 encoded by tumor suppressor BLU on chromosomal 3p region binds Sin 3A and this may contribute to its tumor suppression through modulation of proliferation and apoptosis related gene expression [93, 94]. The repressive potential on gene expression of AML1-ETO/MTG8/ZMYND2 is disturbed when it is fused with RUNX1 on t (8; 21) (q22; q22) translocation, and this facilitates leukemogenesis.

### DEAF-1/ZMYND5 contributes to carcinogenesis through gene expression regulation

The protein ZMYND5 is alternatively called DEAF-1. It contains two conserved domains, the SAND (Sp100, AIRE-1, NucP41/75 and DEAF-1) domain and a cysteine rich MYND domain. Initially SAND was named KDWK after the core of conserved amino acid residues [94, 95]. The DEAF1 transcription factor gene encodes a zinc finger domain-containing protein that functions as a regulator of transcription. Functionally, the encoded product is similar with ZMYND2, 3, 4. SAND domain possesses DNA binding activity.

DEAF-1 is an important transcriptional regulator that is required for development of embryo and clinically linked to depression and suicidal behavior in human individuals [95–97]. It has been shown that deletion of the MYND domain in human DEAF-1 reduces the effectiveness of DEAF-1 in transcriptional repression of promoter of the encoding gene for nuclear ribonucleoprotein A2/B1. DEAF-1 is a binding partner of LMO4 playing an important role in development of mammary gland and genesis of breast cancers [98]. In vitro and in vivo overexpression of DEAF-1 was found to promote proliferation of mammary epithelium [99]; its role in breast cancer occurrence suggests of its activity as an oncoprotein. DEAF-1 has also been known to interact through LMO4 with the tumor suppressor BRCA1, potentially linking it to suppression of breast cancer development [100].

Protein interactions of DEAF1-DEAF-1 and DEAF1-Ku70 are mediated by the SAND domain of the protein DEAF-1 [96], and specific mutations in the domain result in moderate to severe asyndromic intellectual disability in humans. These mutations eliminate or greatly reduce both interactions of DEAF-1 with TTCG-containing DNA sequences and hence its transcriptional repression on its own promoter [97]. DEAF-1 has been shown to be linked to cancer [98, 101], autoimmune disorders [99] and production of interferon-β [100]. In mice DEAF-1 deficiency leads to neural tube closure defects [101]. A nuclear export signal is located with DEAF-1 molecule to act as part of a second interaction domain of DEAF-1-DEAF1 and DEAF-1-LMO4 proteins [102–104].

Other protein–protein interactions are mediated by cysteine rich MYND domain on its C-terminus [105]. The MYND domain within human DEAF-1 spans residues 501–544 as determined by NMR spectroscopy. It is a cysteine-rich module containing a Cys(4)-Cys(2)-His-Cys (C4-C2HC) tandem zinc binding motif, to mediate transcriptional regulatory activity through interactions with cofactors such as N-CoR and SMRT. Similar with the MYND domains within the molecule of AML1/ETO and other ZMYND proteins, the domain DEAF-1 MYND adopts a ββα fold that exhibits tandem zinc-binding sites with a cross-brace topology. It has been shown that the MYND domain of DEAF-1 binds to corepressors SMRT and N-CoR. The binding surface is similar to that previously reported for AML1/ETO/ZMYND2 [106–109].

## ZMYND proteins acting as genomic reader associated with chromatin marks: ZMYND8, and ZMYND11

### ZMYND8 and ZMYND11 recognize and recruit histone marks to regulate gene expression

**Fig. 3:**
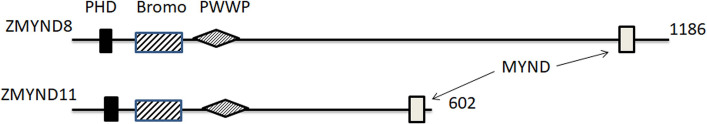
The scheme of motif composition of two ZMYND proteins, RACK7/ZMYND8 and BS69/ZMYND11. They have identical conserved domains with similar activities in histone interactions and genomic reading, and hence implicate in carcinogenesis. The number defines the position of the full length of the molecules with number of amino acid residues, based on individual entry of uniprot. Org, retrieved on September 28, 2020. It has been reported that MYND domain on the C-terminus of ZMYND8 possesses the ability to bind a demethylase, JARID 1D [110]

The proteins RACK7/ZMYND8 and BS69/ZMYND11 are cancer related; they are tumor suppressive or oncogenic with context dependency upon histologic origin. The two proteins have identical motif composition, with plant homeodomain (PHD), BROMO and Pro-Trp-Trp-Pro (PWWP) tandem domain (Fig. 3). These domains function to recognize and bind chromosomal marks, and they both contain a conserved MYND motif function to recognize and bind chromosomal marks [110].

ZMYND 8 was initially identified as an activated binding partner of protein-kinase-C (PKC), and is alternatively called RACK7 [111, 112]. It is a member of the protein family of receptor for activated C-kinase (RACK); molecules of this protein family anchor activated PKC and maintain its increased level of phosphorylation and duration of inactivating state; ZMYND8 contains a PWWP chromatin-binding domain, a bromodomain (BRD), a plant PHD type zinc finger for protein–protein interaction; the PHD–BRD–PWWP (PBP) domains are readers of histone marks to recognize several acetylated and methylated lysine residues, including acetylated lysine 14 of H3 (H3K14ac), H4K16ac, and di- and tri-methylated H3K36 (H3K36me2, H3K36me3) [112–114]. The MYND domain of RACK7/ZMYND8 is mapped on its carboxyterminus spanning the amino acid residues 1028–1062. It has been identified as a transcriptional corepressor; it binds JARID1D known as H3K4 demethylase through interaction mediated by its MYND domain [110]. ZMYND8 is a histone reader mainly targeting to the dual marks H3K4me1 and H3K14ac and that the signature H3K4me1-H3K14ac manifests as a repressive signature for metastasis-linked genes; it has been shown that an anti-invasive factor against prostate cancer metastasis mediates downregulation of metastasis-linked genes [110]. The support of angiogenesis by ZMYND8 has suggested of its tumorigenic potential [115].

The coding gene for the protein BS69/ZMYND11 is harbored in the chromosomal region 10q22. ZMYND11 also contains several histone “reader” modules, including a domain of PHD, a bromodomain, and a PWWP domain, suggesting of its molecular activity involving chromatin regulation; it recruits chromatin mark H3K36 through interaction of PHD-Bromo-PWWP tandem domain [116]. The capacity of ZMYND11 to bind transcription factors C-, B-Myb, MGA, EST2, together with histone modifying enzymes MLL, EZH2, HDAC, KDM3B, NSD1 and corepressor N-CoR has been described (Table 3), [117]. The effect on gene expression regulation exerted by ZMYND 11 remains to be in depth investigation.

**Table 3:** Interacting proteins that BS69/ZMYND11 binds through EFTUD2 binding site

Category of binding partners	Chromatin related factors	Transcription factors
EFTUD2 site binding partner	MLL, EZH2, RNF2/RING1B, PHC3, L3MBTL2, CBX4, BCORL1, HDAC, KDM3B, N-CoR, NSD1	B-Myb, c-Myb, MGA, ETS-2, E2F6, EMSY

The EFTUD2 binding site is located at amino acids 556–562, adjacent to MYND motif at residues 563–598

The chromatin related factors include histone modifying enzymes of methyltransferases (MLL, EZH2, PHC3), demethylases (KDM3B, NSD1), deacetylase (HDAC); E3 ubiquitin-protein ligase (RNF2/RING1B), E3 SUMO-protein ligase (CBX4); corepressors (N-CoR, BCORL1/BCL-6 corepressor-like protein 1); transcription factors include: oncoproteins C-Myb and B-Myb; factor in Myc pathway MGA (MAX gene-associated protein); ETS-2, Transcription factor that activates transcription; E2F6; EMSY, a BRCA2-interacting transcriptional repressor (Based on Ref. [117]).

BS69/ZMYND11 was originally identified as a protein binding adenovirus encoded early antigen E1A and inhibits its transactivation potential [118–120]; The MYND domain on C-terminus of BS69 binds to the PXLXP motif of E1A and Epstein-Barr virus (EBV) determined nuclear protein 2 (EBNA2) [121]. BS69 interacts with EBV encoded transforming membrane integral protein, latent membrane protein 1 (LMP1) through its MYND domain, acting as a scaffold protein in the LMP1-mediated JNK pathway through interacting with a co-factor of tumor necrosis factor (TNF) receptor, TNF receptor associated factor 6 (TRAF6) [122]. The functions of MYND domain within BS69/ZMYND11on ligand binding and molecular interactions have been studied. The interaction between BS69 and its binding proteins of viral and cellular origin requires distinctively charged residues flanking the folded MYND domain, suggesting of a distinctive binding mode; the MYND domain of BS69/ZMYND11 is a conserved zinc binding fold of importance in transcriptional regulation by mediating distinct its molecular interactions with the known viral and cellular proteins [102]. Data also suggests that BS69 downregulates interleukin (IL)-6 mRNA expression induced by NF-κB and LMP1. It has been found that BS69 acts as an endogenous negative regulator of LMP1-induced NF-κB activation by displacing TRADD from the association with LMP1 [123].

### Cancer related ZMYND 8 and 11are abnormally expressed in malignant tissues

RACK7/ZMYND8 has been identified to downregulate metastasis linked genes acting as a transcriptional corepressor collaborating with a histone demethylase. ZMYND8 acts as a reader associating with the dual histone mark H3K4me1-H3K14ac through the PHD/Bromo domain cassette and it has been known the marks H3K4me1-H3K14ac serve as a gene-repressive signature for metastasis-linked genes [124].

**Fig. 4:**
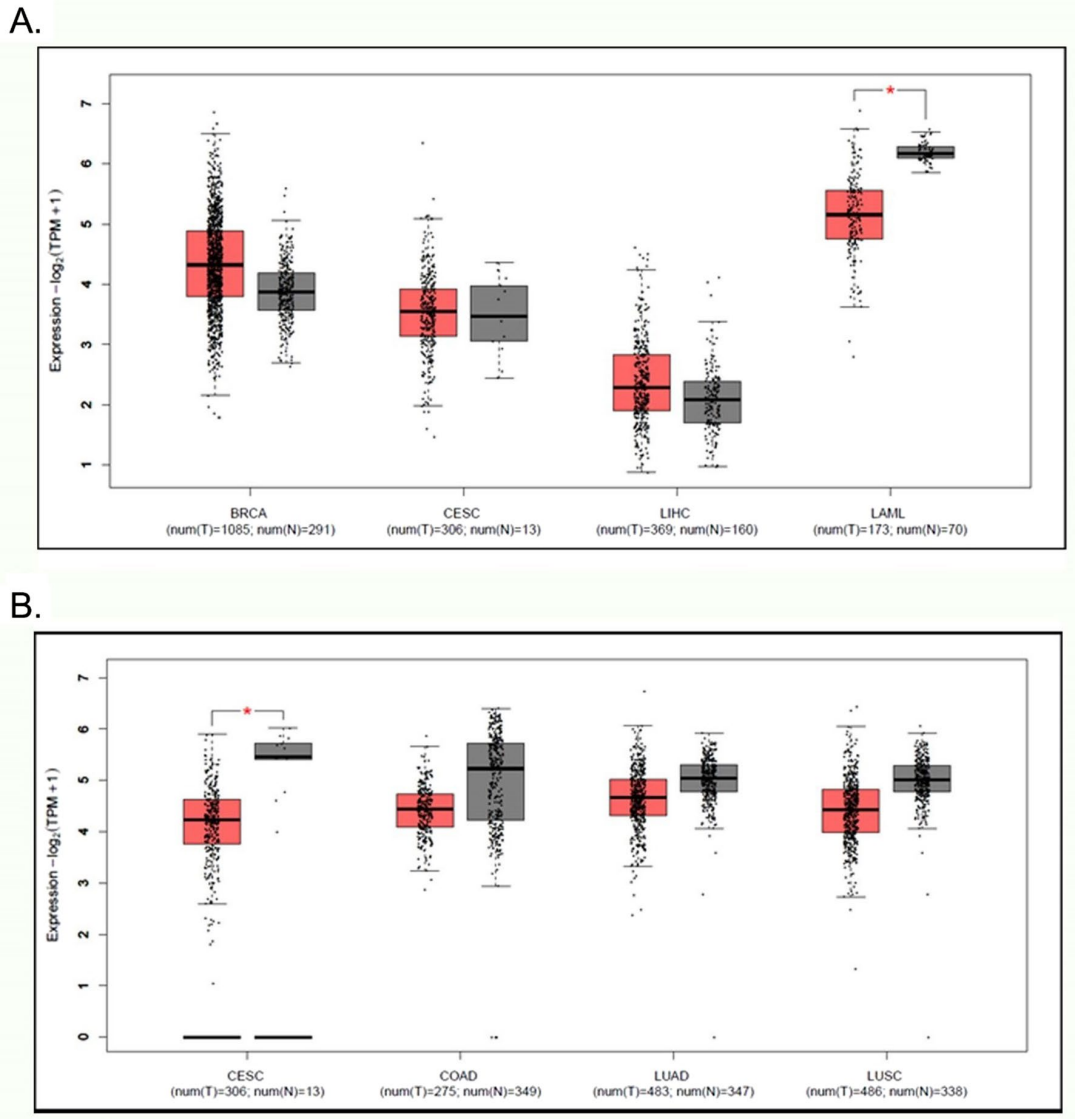
Expression profile of ZMYND8 and 11 in cancer and control normal tissues. **A** The profile of ZMYND8 expression in cancers. The database derived scheme suggests that the levels of ZMYND8 in breast invasive carcinoma (BRCA) and hepatocellular carcinoma (LIHC) are elevated, while in cervical squamous cell carcinoma (CESC) the level is not different in malignant versus normal tissue; in AML (LAML) the expression level of ZMYND8 is remarkably downregulated (*p < 0.05). **B** The scheme of ZMYND11 expression with a new one with profile in different cancers. The expression of ZMYND11 is significantly downregulated in cervical carcinoma (*p < 0.05), and also slightly downregulated in colon adenocarcinoma (COAD), lung adenocarcinoma (LUAD), and lung squamous cell carcinoma (LUSC) (http://gepia2.cancer-pku.cn/#analysis, retrieved on November 21, 2022)

ZMYND8 is implicated in occurrence of cancers in a manner dependent on tumor typeI; its expression level is varied among different type of tumors (Fig. 4). It contributes to either carcinogenesis or tumor suppression. In breast cancer and prostate cancers, ZMYND8 has been reported as a tumor suppressor that cooperates with the histone demethylases, KDM5 family functions to remove methyl group from the mark H3K4me3, such as KDM5C [110, 125]. It has been reported that ZMYND8 is overexpressed at protein and mRNA levels in considerable amount of hepatocellular carcinoma (HCC) cases. High level of ZMYND8 expression has been shown to be significantly correlated with microvascular invasion, high Edmondson grade, and elevated level of alpha-fetoprotein. Overexpression of ZMYND8 mRNA has been suggested as an independent prognostic factor predicting early recurrence and short recurrence-free survival (RFS) [126]. It has been shown that, however, upregulation of ZMYND8 by hypoxia-inducible factor (HIF) or ZMYND8 overexpression via positive feedback of the estrogen receptor (ER) pathway was correlated with poor prognosis in patients of breast cancer [127]. These findings reveal that ZMYND8 is oncogenic.

ZMYND8 is essential for AML proliferation in vitro and in vivo. It has been shown that ZMYND8 associates with oncoprotein MYC and interferon regulatory element 8 (IRF8) enhancer elements in cell lines and in patient samples. ZMYND8 occupancy at IRF8 and MYC enhancers requires a transcription coactivator BRD4 which is also necessary for AML proliferation. ZMYND8 binds to the ET domain of BRD4 via its chromatin reader cassette, which is required for proper chromatin occupancy and maintenance of leukemic growth [128].

27-Hydroxycholesterol (27-HC) is the most abundant oxysterol that increases the risk of breast cancer progression. Data from mouse mammary tumor and human breast cancer models show that the ZMYND8 acting as histone reader was selectively expressed in breast cancer stem cells (BCSCs) and promotes epithelial-mesenchymal transition (EMT). Mechanistically, ZMYND8 was a transcriptional regulator of 27-HC metabolism. It increased cholesterol biosynthesis and oxidation but blocked cholesterol efflux and 27-HC catabolism, leading to accumulation of 27-HC in BCSCs. The results obtained support that 27-HC promoted EMT, oncogenic transformation through metabolic reprogramming [129]. Cholesterol metabolism contributes to the genesis of colorectal cancer (CRC). The effect of the inhibitor of cholesterol biogenesis mevalonate (MVA) pathway has been investigated. YAP-mediated ZMYND8 expression sensitizes intestinal tumors to the inhibition of the pathway. The oncogenic activity of YAP relies largely on ZMYND8 to enhance intracellular de novo cholesterol biogenesis [130].

It has been known that ZMYND8 also exerts tumor suppression. It has been demonstrated that (ZMYND8 suppresses stemness, drug resistance, and tumor-promoting genes, which are hallmarks of cancer. The ability of ZMYND8 to chemo-sensitize doxorubicin-treated metastatic breast cancer cells by downregulating tumor-associated genes was further confirmed. Overexpression of ZMYND8 in doxorubicin-treated cells stimulated those involved in a good prognosis in breast cancer. And ZMYND8 modulates the bivalent or poised oncogenes through its association with KDM5C and EZH2, thereby chemo-sensitizing the cells to chemotherapy for better disease-free survival [131].

Perturbation of ZMYND8 promotes initiation and progression of cancers. In fact, ZMYND8 is an antigen associated with cutaneous T-cell lymphoma [132]. In addition, chimeric transcripts of ZMYND8-v-rel reticuloendotheliosis viral oncogene homolog A (avian) (RELA) were reported in a pediatric patients with acute erythroid leukemia, known as a type of AML [133]. The fused gene constitutively activates NF-κB in AML cells, as the RELA gene is a member of NF-κB family. The formation of the fused enables NF-κB 3/p65 to be under the control of the ZMYND8 promoter [133].

In breast cancer cells, ZMYND8 is expressed as a fusion protein with centrosomal protein 250 (CEP250), a factor required for centriole–centriole cohesion during interphase of cell division [134]. In mismatch repair-deficient colorectal cancers (CRC) ZMYND8 has a mutation rate of 19% [135]. The gene ZMYND8 is located within a chromosomal region with recurrent alterations of somatic copy number in high-grade serous ovarian cancer [135]. An increased copy number (two to six copies) of ZMYND8 has been identified in prostate cancer derived cells [136].

A recurrent chromosomal translocation t(10; 17)(p15; q21) in-frame fuses an N-terminal gene segment of coding gene for ZMYND11, with the entire coding region of Malignant Brain Tumor domain containing 1 (MBTD1) and generates an abnormal chimeric gene named ZM [115, 137, 138]. It is detectable among a subset of AML patients. Clinically, a minimally differentiated cell phenotype is displayed in ZM-positive AML cases and poor prognosis-related gene markers (such as HOX cluster genes) are expressed, indicative of adverse outcome [139]. The recurring chromosomal translocation t(10;17)(p15;q21) involving coding gene of ZMYND11 is present in a subset of human acute myeloid leukemia (AML) patients. It has been shown that ZM confers primary murine hematopoietic stem/progenitor cells indefinite self-renewal capability ex vivo and causes AML in vivo. It has also been revealed that ZM directly binds to and maintains high expression of pro-leukemic genes including Hoxa, Meis1, Myb, Myc and Sox4, and recruits the NuA4/Tip60 histone acetyltransferase complex to cis-regulatory elements, sustaining an active chromatin state enriched in histone acetylation and devoid of repressive histone marks. The essential requirements of Tip60 interaction and an H3K36me3-binding PWWP (Pro-Trp-Trp-Pro) domain for oncogenesis have been demonstrated [140].

The MYND domain of BS69/ZMYND11 recruits co-repressors such as N-CoR and HDAC complexes [141]. Its implication in carcinogenesis is manifested to be dependent on a tissue origin; it has been reported that it has a copy number variation (CNV) in acute myeloid leukemia [142]. Data exploited from mining, as well as our experimental observation revealed that ZMYND 11 is downregulated in solid cancers, notably in ovarian cancer (Fig. 4).

## ZMYND proteins that contain only MYND domain with less characterized functions: ZMYND7, ZMYND9, ZMYND10

### ZMYND proteins with similar structure but variant biological activities

Among members of the ZMYND family, programmed cell death 2 (PDCD2)/ZMYND7, ubiquitin specific protease 19(USP19)/ZMYND9, and beta catenin in lung cancer (BLU)/ZMYND10 only contain MYND motif in their respective carboxylterminus, with length between 30 and 40 amino acid residues. The alignment of sequence using online software Clustal reveals a similarity between USP19/ZMYND9 and BLU/ZMYND10 greater than that between PDCD7/ZMYND7 and BLU/ZMYND10 (Fig. 4). The position and length of the MYND motif of the proteins are indicated in Table 4. They have distinctively different, and less characterized biological functions. PCDC2 is a proapoptotic molecule implicating in the development and maturation of lymphoid organ, similar with the protein of the PDCD family, PDCD 5 [143]. It has also been known as a TSG [144].

**Table 4:** Motif composition of PDCD2/ZMYND7, USP19/ZMYND9, BLU/ZMYND10

Proteins/parameters	ZMYND7	ZMYND9	ZMYND10
Full length	344	1318	440
CS 1	–	113–202	
CS 2	–	282–384	
USP		497–1214	
MYND	135–172 (38)	791–833 (43)	394–430 (37)

The linear structure shows that USP19/ZMYND9 contains additional functional regions C1, C2 and USP which possesses catalytic activity of ubiquitin protease. The numbers denote the position of amino acid residues, and those in brackets showing the length of the fragment. The three proteins share the domain of ZMYND

USP19/ZMYND9 is a deubiquitinase, capable of stabilizing the KPC1 (Kip1 ubiquitylation-promoting complex 1) ligase for p27Kip1, c-IAP1, and inhibiting the degradation of an anti-apoptotic molecule cellular inhibitor of apoptosis (cIAP) [145]; while it has been reported that BLU/ZMYND10 which engages intracellular NF-κB pathway to downregulate its transcriptionally activated factor like cIAP2 [17]. USP19/ZMYND9 also interacts with HIF-1α and several components of the hypoxia pathway to inhibit their degradation in a deubiquitinase independent manner [146–148]. It has been found to positively regulate autophagy; it removes K11-linked ubiquitin chain of the autophagy executor Beclin-1 at the residue lysine 437 and hence stabilizes Beclin-1. USP19/ZMYND9 also negatively regulates signaling pathway of type I interferon (IFN) with dependence of Beclin-1 [149]. There is data showing that the level of USP19 serves as an indicator of clinical outcome of breast cancer; its implication in carcinogenesis, however, remains to be further characterization [150].

### BLU/ZMYND10 has been shown to be downregulated in tumors, with tumor suppression to be mechanistically characterized

BLU is mapped on the fragment LUCA (deleted in lung cancer) of region p21 on the short arm of human chromosome 3 (3p21). It was initially identified as a frequently deleted fragment in lung cancer; the term BLU was derived from beta-catenin like fragment in lung cancer [151]. Subsequently it was discovered that BLU is inactivated in a variety of human cancers through different approaches including genetic deletion and epigenetic silencing. The silence of BLU has also been described in, glioma [152], gallbladder cancer [153], cervical cancers [154], and NSCLC [155].

The coding product of BLU is termed ZMYND10, which is a ZMYND protein with a MYND domain with a length of 34 amino acid residues [13]. The expression of ZMYND10 has been found to absence in 20% of hepatoma cases [156], and a number of esophageal squamous cell carcinoma (ESCC) [157]. NPC has been known as a tumor endemic with certain regions in the world, including southern China and Southeast Asia [158]. In Asian NPC, loss of homozygosity (LOH) is frequently detected at several chromosomal regions, particularly the locations 3p, 9p, 11q, 13q and 14q [159–164]. It has been reported that the anomaly in 3p is among key changes during occurrence of NPC [159]. A high frequency of LOH in 3p was also found in normal nasopharyngeal epithelial cells and precancerous lesions in individuals from endemic areas, suggesting that the inactivation of TSGs in this chromosome might be an early event in the genesis of NPC [159]. Coding genes for p16/CDKN2A and RASSF1A on 9p and 3p respectively have been recognized as main TSGs in NPC [159–164]. Mutability of RASSF1A has been reported in NPC [165]. CCND1 coding for cyclin D1(cycD1) has been observed to be amplified in NPC epithelium; the role played by cycD1 in EBV mediated transformation has been documented [165].

The inactivation of BLU/ZMYND10 due to promoter hypermethylation has been observed in clinical specimens, as well as passaged cell lines of NPC. Its promoter hypermethylation was detected in the primary tumors, but not in normal nasopharyngeal epithelium [166, 167]. The expression of BLU is correlated to a favorable prognosis, prolonged survival of NPC patients [168]. It had been reported that BLU is expressed in a stress-responsive manner, in NPC tumors the expression is disrupted by either epigenetic or genetic mechanisms [167]. How BLU coding protein regulates downstream biological events, remains to be elucidated.

Several tumor suppressor proteins with zinc finger of different types within the molecule have been found to regulate gene expression by engagement of intracellular signaling pathways. It has been reported that ZNF 569, with a KRAB and ZNF domain of zinc finger, is a protein expressed in most of the examined human adult and embryonic tissues; it level is higher in muscle of heart and skeleton. In COS-7 cells ZNF569 inhibited the activities of transcriptional regulation by SRE and AP-1 similar with the activity of siRNA against ZNF569. The data also suggest that it represses gene transcription to suppress MAPK signaling pathway, so as to mediate cellular functions [169]. Another molecule, ZNF411 as member of the same zinc-finger protein family, has also been recognized as a transcription factor, working as a negative regulator in MAPK signaling pathway [170].

Based on structural similarity, it had been speculated that ZMYND10 exerts its biological activities through engaging intracellular pathways and hence regulating gene expression. We have shown that re-expression of BLU in NPC, esophageal cancer derived cells inhibits the pathways of JNK and ERK of MAPK family, downregulating the kinase catalyzing protein phosphorylation modification [16]; the axis of MAPK-cyclin D was inhibited, and the cells arrested at G1 phase. The tumor suppressor gene on 3p21, RASSF1A, suppresses tumor formation through negative regulation of oncogene Ras [171], and it has been recognized as the key tumorigenic gene in NPC on mutational or epigenetic inactivation. We have observed that similarly with RASSF1A, BLU/ZMYND10 interferes the activity of mutant Ras to prevent cancer cell proliferation by inhibiting MAPK/ERK signaling [172].

Proteins of ZMYND family with tumor suppressive potential antagonize occurrence of tumors by repressing gene transcription; the activity is achieved by association of repressors, like MRT, N-coR or SIN 3A or SIN3B. The molecular interactions with the co-repressors in context of leukemogenesis have been extensively studied in ZMYND 2, 3 and 4, encoded by genes which are fused with molecules with transcription factors [80, 82, 83]. We have recently described that ZMYND10 is associated with SIN3A when re-expressed in a hepatoblastoma line HepG2 [94, 96]. The implication of such binding in the genesis of the liver cancers remains to be characterized, the data, however, supports that BLU/ZMYND10 contribute to suppressing tumor formation through transcription repression.

ZMYND proteins which are recognized to be tumor suppressors in both hematopoietic and solid neoplasms, ZMYND8 and 11 possess functional domains to recognize histone code or recruit histone modifying enzymes, for example lysine demethylases. ZMYND8 inhibits migration of breast cancer cells through genomic reading activity of recognizing histone mark [38]. And the ZMYND11 protein with reading ability of an active mark H3K36 has been shown copy number variation (CNV) in certain type of lymphoma, suggesting its possible role in tumorigenicity [142]. We have recently reported that the expression of BLU/ZMYND10 induces a repressive histone mark trimethylated lysine 9 of histone 3 (H3K9me3) in lung cancer line H1299 or hepatoblastoma HepG2, both cell lines are absent of BLU expression during in vitro culture [95]. It had been shown that methylated H3K9 is recruited to the promoter region of CCND1 coding for cyclin D1 [38]. In H1299 cells with elevated H3K9me3, the expression level of G1 cyclin, cyclin D1 was downregulated correlated with the altered cell cycle profile, that the cells were arrested at G1 phase; in HepG2 cell ectopically expressing BLU/ZMYND10, the elevated H3k9me3 level was correlated with concurrently reduced cyclin D1 and a G2 phase cyclin, cyclin B1, and the cells were arrested at G1 and G2 phases [95]. The data suggests that a candidate tumor suppressor BLU/ZMYND10 engages intracellular signaling pathway, associates with components of co-repressor complex, and induces repressive histone mark through a possible interaction with modifying machinery of histone code, to downregulate gene expression so as to inhibit cell cycle progression, and potentiate apoptosis and hence contribute to tumor suppression.

These findings raised the possibility that BLU/ZMYND10 targets to some histone modifying enzymes to alter the level of some modified epigenetic codes on peptide tail of H3. While global screening of how multiple epigenetic codes act on ZMYND10 mediated tumor suppression remains to be done, reversing of H3K9 demethylation by ZMYND10 leading to repressing expression of cell cycle regulator has been suggested, as supported in part by some published work [38]. H3K9 is specifically modified by a group of demethylase of lysine, termed KDM4, some of which have been known as oncopoteins. A member of the family, KDM4C, alternatively called gene activated in squamous cancer 1(GASC1). It functions to demethylate methylated H3K9 so as regulate architecture of chromatin and gene expression [173–176]. A transformed phenotype including increased proliferation, anchorage-independent growth, growth factor independent proliferation, disorganization of cellular architecture, and increased mammosphere formation has been observed in nontransformed mammary epithelial cells overexpressing GASC1 [177]. Its expression has been shown to be correlated with the outcome of ovarian cancer [178]. Study in ESCC suggests that a significant association with lymph node metastasis (P = 0.030) and tumor-node metastasis stages (P = 0.013) was noted in cases with its nuclear expression. Analysis with Kaplan–Meier survival analysis revealed a tendency with poor survival of ESCC patients associated with high expression of GASC1 [179].

Accumulating evidence suggests the cancer drivers KDM4A, KDM4B, and KDM4C are overexpressed leading to the efficient growth, in a variety of human malignancies including breast, colorectal, lung, prostate, and other cancers. Among them amplification KDM4A and KDM4B are observed in gastric cancer, neuroblastoma, and ovarian cancer [178, 180–182]. KDM4A interacts with the co-repressor N-CoR to suppress the TRAIL-DR5 pathway and KDM4A meanwhile functions as a key regulator in metabolism of tumors via E2F114 [183]. KDM4B plays a role in DNA repair and in triggering of mitochondria dependent apoptosis, and reprograms the genomes to control cell arrest in somatic cells isolated from cloned embryos [184]. The activities of KDM4A-4C to coactivate androgen receptor and estrogen receptor, imply of their application as promising epigenetic drug targets [185–189].

Our working hypothesis on the mechanism of BLU/ZMYND10 in repressing gene expression is that it binds some modifying enzymes notably histone demethylases and blocks their activity so as to increase the level of the repressive mark(s) on histone 3 which is mostly regulated by modification with methylation.

## Conclusion

The proteins of the ZMYND family are highly conserved among species, and are involved in embryonic development as well as carcinogenesis. More than 20 members identified to date contain a common zinc finger- MYND motif, together with different functional domains responsible for molecular interactions like binding and recruitment of active molecular to target sequence, association with transcription regulators, mainly repressors, and catalyzing modification of histone and nonhistone proteins. It has been known that the proteins exert oncogenic potential or tumor suppression during the occurrence of tumors. Precise elucidation of their activity would lead to their utilizition as anticancer therapeutic targets, in complementary to the routine treatment modalities.

## Data Availability

Non-applicable.

## Abbreviations

DEAF-1: Deformed epidermal autoregulatory factor-1
ESCC: Esophageal squamous cell carcinoma
EZH2: Enhancer of zeste homolog-2
MYND: Myeloid Nervy deformed auto-regulatory factor-1 containing
SET: Suppressor of variegation, enhancer of zeste, trithorax
NPC: Nasopharyngeal carcinoma
NSCLC: Non-small cell lung cancer
SMYD: SET and MYND containing motif
TNBC: Triple negative breast cancer
ZMYND: Zinc finger MYND containing motif
