# Correction: Zinc finger myeloid Nervy DEAF-1 type (ZMYND) domain containing proteins exert molecular interactions to implicate in carcinogenesis

**DOI:** 10.1007/s12672-023-00640-3

**Published:** 2023-04-26

**Authors:** Longji Wu, Jing Huang, Pankaj Trivedi, Xuerong Sun, Hongbing Yu, Zhiwei He, Xiangning Zhang

**Affiliations:** 1 Department of Pathophysiology, Guangdong Provincial Key Laboratory of Medical Molecular Diagnostics, School of Basic Medicine, Guangdong Medical University, Songshan Lake Scientific and Industrial Park, Dongguan, 523808 Guangdong People’s Republic of China; 2 Guangdong Provincial Key Laboratory of Medical Molecular Diagnostics, Chinese-American Tumor Institute, Guangdong Medical University, Dongguan, Guangdong People’s Republic of China; 3 Guangdong Provincial Key Laboratory of Medical Molecular Diagnostics, Institute of Aging, Guangdong Medical University, Dongguan, Guangdong People’s Republic of China; 4 Department of Experimental Medicine, La Sapienza University, Rome, Italy; 5 Present Address: Institute of Modern Biology, Nanjing University, Nanjing, Jiangsu China

**Correction: Discover Oncology (2022) 13:139** 10.1007/s12672-022-00597-9

After publication it was noticed that the online derived data within Fig. 4B was controversial with what was obtained from the ongoing experiments. Due to this, the following corrections have been made:

1. An updated version of Figure 4 is available, the old (Fig. 1) and new figure (Fig. 2) are shown in this correction article.

**Fig. 1:**
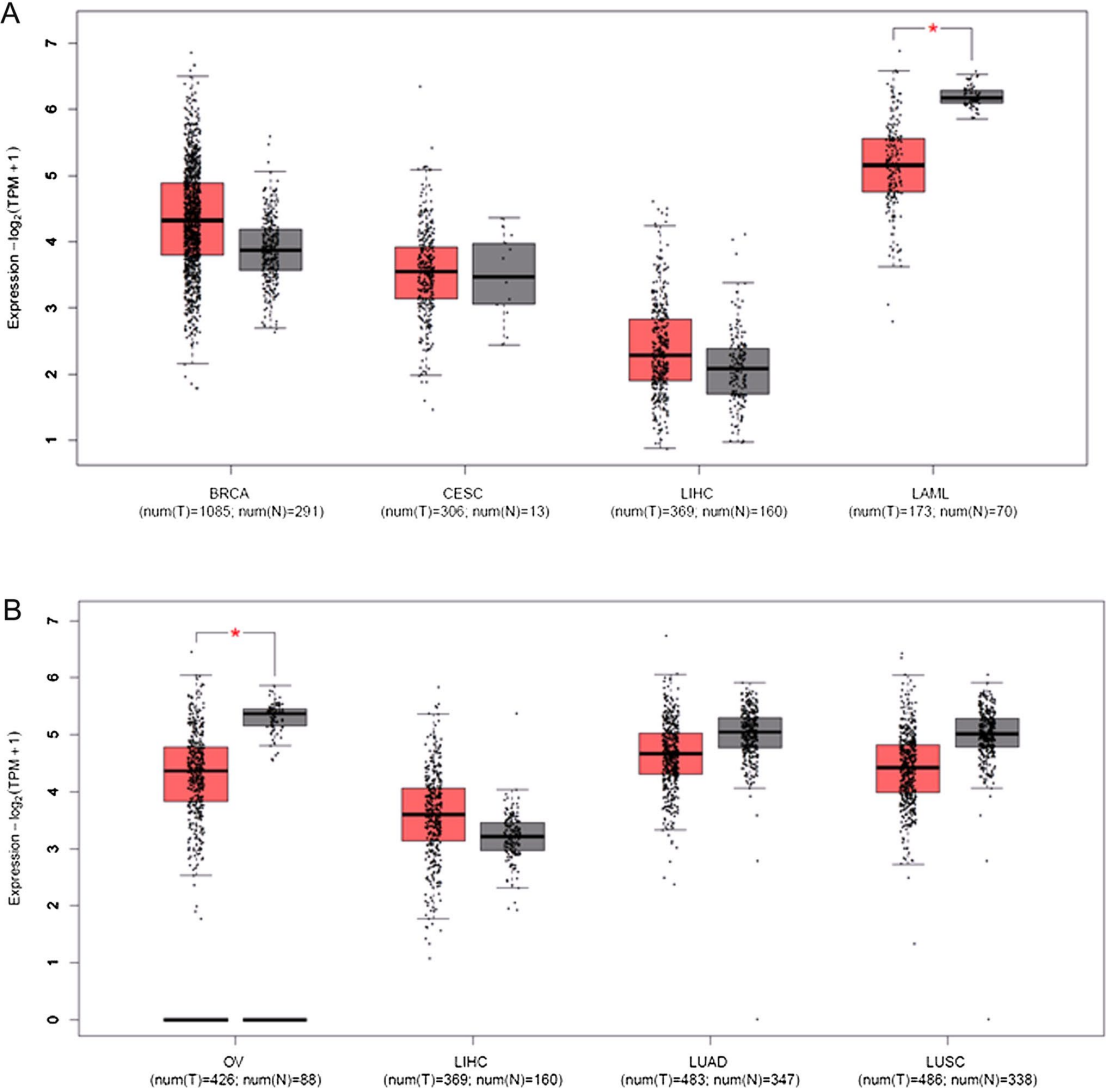
Original version of Figure 4

**Fig. 2:**
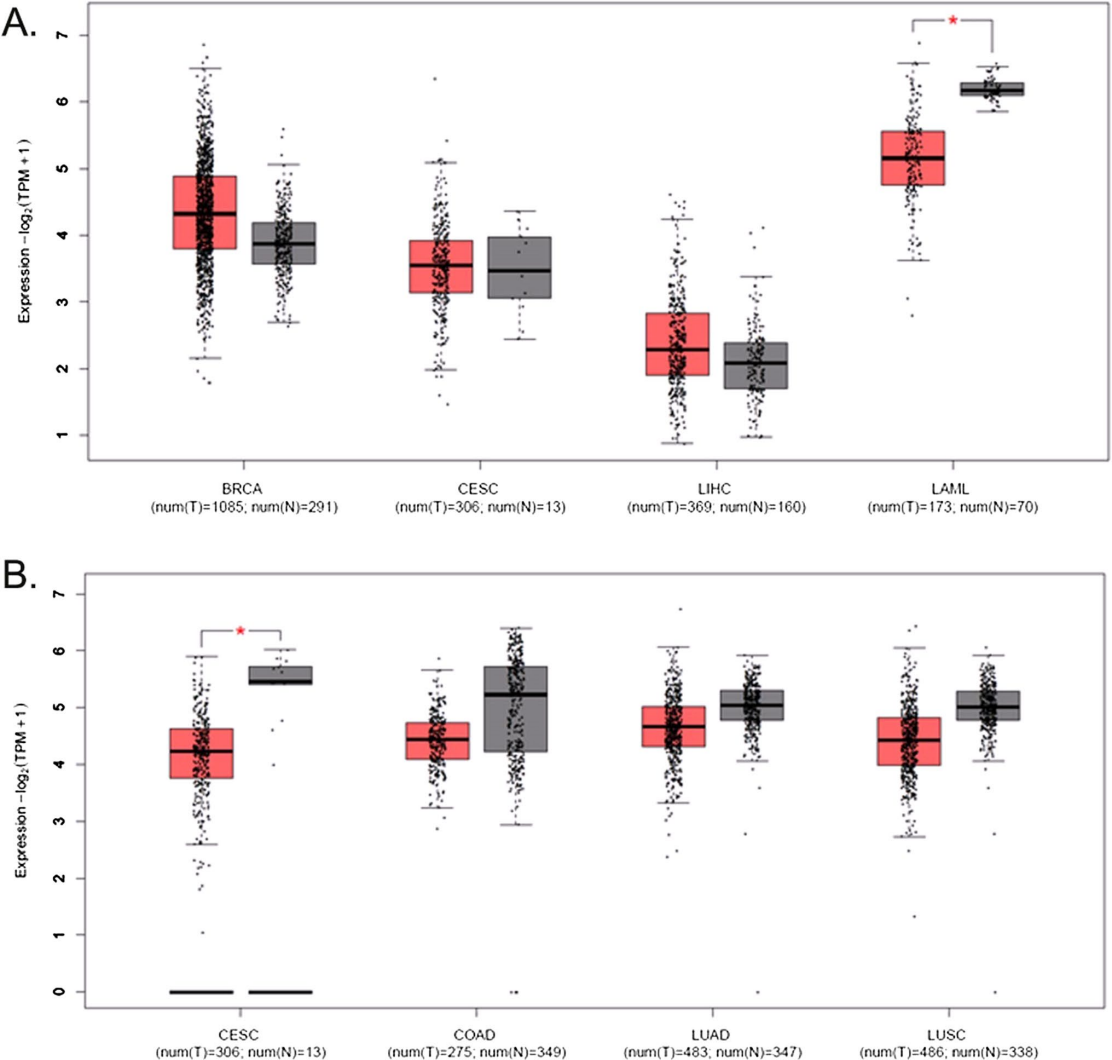
Updated version of Figure 4
2. The last sentence under the paragraph “4.2 Cancer related ZMYND 8 and 11” has been changed from:
  - “**revealed that ZMYND 11 is downregulated in solid cancers, notably in cervical cancer (Fig. 4)**”
    To:
  - “**revealed that ZMYND 11 is downregulated in solid cancers, notably in ovarian cancer (Fig. 4)”.**

Finally author H. Yu is now listed as a corresponding author. These changes do not affect the conclusions of the article. The original article [1] has been updated.
